# When Should We Perform Endoscopic Drainage and Necrosectomy for Walled-Off Necrosis?

**DOI:** 10.3390/jcm9124072

**Published:** 2020-12-17

**Authors:** Tanyaporn Chantarojanasiri, Thawee Ratanachu-Ek, Hiroyuki Isayama

**Affiliations:** 1 Department of Internal Medicine, Rajavithi Hospital, Ministry of Public Health College of Medicine, Rangsit University, Bangkok 10400, Thailand; 2 Department of Surgery, Rajavithi Hospital, Ministry of Public Health College of Medicine, Rangsit University, Bangkok 10400, Thailand; thawee1958@gmail.com; 3 Department of Gastroenterology, Graduate School of Medicine, Juntendo University, Tokyo 113-8421, Japan; h-isayama@juntendo.ac.jp

**Keywords:** walled-off pancreatic necrosis, endoscopic drainage, necrosectomy

## Abstract

Endoscopic drainage and necrosectomy are now accepted treatment approaches for patients with symptomatic walled-off pancreatic necrosis (WON). The current recommendations advocate step-up approaches for the treatment of symptomatic WON. Previous recommendations stipulated that endoscopic intervention should be delayed until more than four weeks after the onset. Recent data on early drainage have been increasing and this option might be considered in well-encapsulated cases, but the percutaneous route is preferred if the drainage is performed within two weeks after onset or in nonencapsulated cases. Recently, additional drainage methods, such as the multiple gateway technique and multiple stent placement, have been developed to open up multiple dead spaces in the WON cavity. Endoscopic necrosectomy could be performed via the transluminal route or percutaneous route after failed initial and additional drainage procedures. The use of novel lumen-apposing stents is a promising treatment option that could reduce the number of steps, the procedure time, and the overall number of necrosectomies.

## 1. Introduction

Pancreatic fluid collection (PFC) is one of the local complications that occurs after acute pancreatitis. Recently, the gold standard for management of pancreatic fluid collection has changed from aggressive debridement to a more conservative approach. Endoscopic treatment has been accepted as the standard treatment for this condition. However, the timing of endoscopic treatment was adopted from data collected using other approaches. With increasing data regarding the endoscopic treatment, the optimal timing for the procedure has been reconsidered. This review summarizes the data emphasizing the timing of endoscopic and other approaches for pancreatic walled-off necrosis drainage as well as endoscopic necrosectomy. To achieve this, a search was made of English-language human studies listed in the PubMed database, EMBASE, and others that were published between 2007 and November 2020. The following keywords were used alone or in combination with pancreatic walled-off necrosis: necrotizing pancreatitis, timing, early drainage, percutaneous drainage, surgical drainage, endoscopic drainage, necrosectomy, step-up approach, stents, lumen-apposing stents, and multigateway. The references of identified articles were also searched for potentially relevant studies. Systematic reviews, meta-analyses, and case reports of special techniques were included. Duplicated data or data published as abstracts in academic meetings were excluded.

## 2. Evolution of Pancreatic Fluid Collection

**Figure 1:**
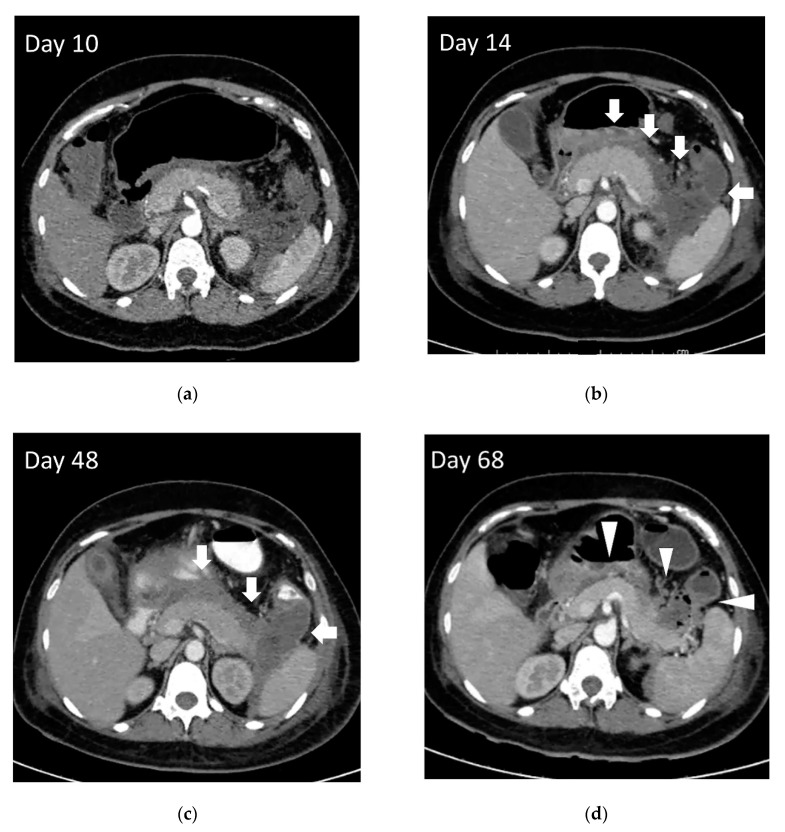
The evolution of the pancreatic necrosis and well-encapsulated walled-off necrosis. (**a**) The encapsulation was not completed 10 days after onset. (**b**,**c**) The peripancreatic fluid collection formed an encapsulation (arrow) within two weeks of the onset. (**d**) This patient developed infected walled-off necrosis with a cavity containing air bubbles (arrowhead) that needed drainage at day 68 after the onset of pancreatitis.

Based on the pathophysiology, acute pancreatitis can be divided into two types: interstitial edematous pancreatitis and necrotizing pancreatitis [1]. The edematous inflammations consist of pancreatic fluid leakage that then forms a peripancreatic fluid collection and develops into a pancreatic pseudocyst, while the necrotic collection forms into acute necrosis and later becomes a walled-off necrosis [1] (Figure 1). Most patients with interstitial pancreatitis have mild symptoms that resolve within one week [2]. On the other hand, 20% of patients will develop necrotizing pancreatitis, which later will turn into walled-off necrosis [3]. These patients usually have a more severe condition associated with higher rates of organ failure, ICU stay, and mortality [2].

## 3. Treatment of Walled-Off Necrosis

### 3.1. Indications for Walled-Off Necrosis Drainage

The well-accepted indications for pancreatic necrosis drainage are ongoing organ failure, infection, organ compression, and compartment syndrome [4]. In the case of sterile necrosis, drainage and debridement might be indicated in cases with pain, nausea/vomiting, nutritional failure, fistula, persistent inflammation, or local compression [5]. The drainage aims to remove the infected debris, relieving internal pressure and reducing systemic inflammatory response [6].

Earlier approaches to infected acute necrosis collection were performed by percutaneous or surgical drainage. Surgical drainage provides more efficient removal of infected debris. The percutaneous approach provides some degree of drainage and an access route into the infected cavity with less invasiveness. However, it might not be sufficient in severe cases [5]. Recently, endoscopic guided transmural drainage has been accepted as a standard of care for patients with walled-off necrosis [7]. The endoscopically created tract could be used as a portal for endoscopic debridement, especially when the newly developed devices are used. The indications for necrosectomy are shown in Table 1.

### 3.2. Timing for Treatment of Walled-Off Necrosis

The timing for initiating drainage for pancreatic fluid collection has changed over time. Initial studies where early and aggressive surgical intervention was performed showed higher morbidity and mortality when compared with delayed necrosectomy [8]. Recent recommendations in the management of walled-off necrosis defer the catheter drainage of pancreatic necrosis until the walled-off process has been reached, which usually takes around four weeks [9].

### 3.3. Step-Up Approach

The concept of the step-up approach is to treat the patient conservatively and start with less invasive procedures. The strategy is to perform percutaneous or endoscopic transluminal drainage and proceed to further treatment if the patient does not clinically improve within 72 h. If the initial drainage fails, multiple sites of percutaneous drainage might be considered, followed by video-assisted retroperitoneal debridement (VARD) [10] or transluminal necrosectomy. The timing of the first intervention ranges from 11 to 155 days in the RCT study, but these data are based on necrosectomy performed by laparotomy or VARD [11].

#### 3.3.1. Endoscopic Step-Up Approach

Endoscopic guided placement of cystogastrostomy or cystoduodenostomy stents, depending on the access route, is performed, followed by endoscopic necrosectomy if the symptoms do not improved [12]. In many cases, adjunctive irrigation with a nasocystic drainage tube was used [13]. Apart from the endoscopic approach, combined endoscopic–percutaneous approaches are recommended, especially in cases where the collection extended beyond endoscopic reach [14]. Moreover, having multiple endoscopes in different locations, the so-called “multigateway approach,” is employed to maximize the drainage ability by using either multiple plastic stents [15] or multiple LAMS [16]. In cases with multiple subcavities, multiple plastic stents could be placed between the gastric lumen and small cavity through the connection with the main cavity [17]. These additional drainage methods aim to open up multiple dead spaces in the walled-off pancreatic necrosis (WON) cavity that are causes of persistent infection. Through this endoscopic step-up approach, many invasive procedures can be avoided, which should lead to reductions in hospital stays, morbidity, and mortality [11].

#### 3.3.2. Percutaneous and Surgical Drainage with Step-Up Approach

In patients where early drainage is indicated, the percutaneous route is still preferred over endoscopic transmural drainage because well encapsulation takes time to develop [5]. The timing of percutaneous drainage was reported to range from one to 154 days after the onset of pancreatitis [18,19]. The patients were evaluated 72 h after the procedure before proceeding with step-up treatments. According to a systematic review, early percutaneous drainage treatment seems to reduce the need for surgical necrosectomy due to improved control of pancreatic fluid leakage [19]. The complications after percutaneous drainage (PCD) were reported to be similar when performed within four weeks, when the lesion was still not encapsulated, or later than four weeks [20]. In 33% of patients who received PCD as a step-up approach, further necrosectomy was required [11].

Surgical treatment was once considered the standard of care for pancreatic necrosis. The timing for surgical intervention has changed from 72 h to more than 30 days or as late as possible [21].

## 4. Endoscopic Drainage

Endoscopic treatment for peripancreatic fluid collection has been used since 1975 for direct transluminal puncture and aspiration [22]. The procedure has shifted from endoscopically guided simple aspiration or fistulotomy to endoscopic ultrasound-guided drainage [23]. By placing a stent over the newly created tract, the necrotic fluid and debris can be drained into the luminal cavity and vice versa. For safe drainage without free peritoneal perforation, effective encapsulation of the collection is warranted. While a cutoff point of four weeks was estimated for the walled-off formation, full encapsulation could be seen in up to 43.3% of patients [24]. The timing of endoscopic drainage was adopted from the data using other interventions—that is, more than four weeks after the onset of acute pancreatitis [10]. However, in many cases, the indication for drainage occurs earlier and percutaneous intervention is generally recommended in such situations [5]. On the other hand, in cases where a lesion is located in the central area of the retroperitoneal region, it is much easier to approach by endoscopy, so endoscopic drainage might be performed after the encapsulation is confirmed [25].

There have been a few retrospective studies of early endoscopic drainage in walled-off necrosis. In one study, in a series of direct endoscopic necrosectomies using metallic stents, no procedure-related complications were reported. Another two comparative studies between early (<4 weeks) and delayed conventional drainage also showed no increase in morbidity or mortality if the procedure was performed in an encapsulated cavity [24]. The median time for early drainage in these retrospective studies was 19 to 23 days after the onset of acute pancreatitis [26]. Complications such as perforation or bleeding did not significantly increase in patients who received early drainage [25].

### 4.1. SEMS as an Adjunctive Strategy to Improve Endoscopic Drainage

**Figure 2:**
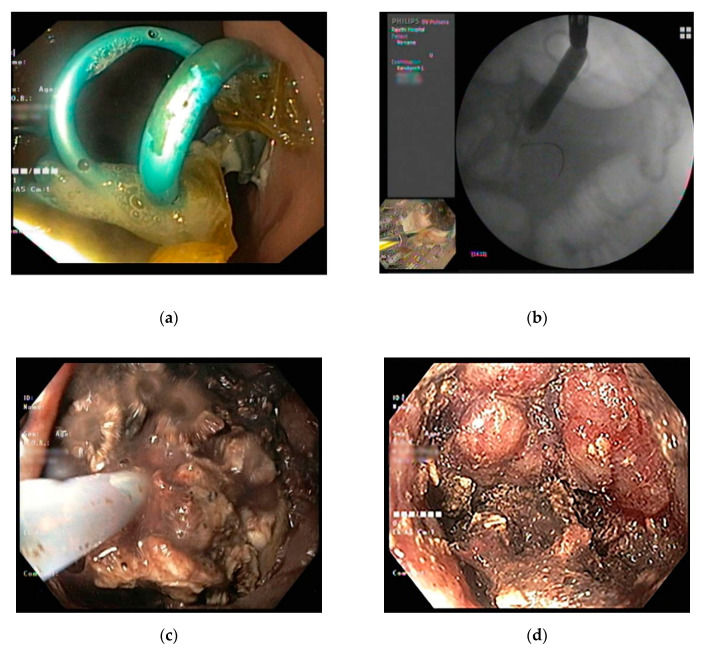
(**a**) Endoscopic necrosectomy after EUS-guided placement of multiple plastic stents. (**b**) After stent removal, the puncture site is dilated using a balloon and the scope is inserted into the cavity. (**c**) The debris is removed by irrigation and mechanical removal until (**d**) pink granulation tissue is seen.

The benefits of endoscopic drainage include lower invasiveness and good proximity to the retroperitoneal region. However, the access portal size is still the main limitation. In the case of walled-off necrosis, the tissue debris cannot be drained through multiple pigtail stents so additional procedures are usually needed (Figure 2). Before the development of dedicated stents for pancreatic fluid collection drainage, fully covered self-expandable metallic stents (FCSEMS), either biliary or esophageal, were used to aid the endoscopic removal of tissue debris [26,27,28]. In reports using esophageal FCSEMS with a diameter of 18 to 20 mm, total necrosectomy could be achieved within three sessions of endoscopic necrosectomy [27,28]. However, major complications such as migration and occlusion occurred [28]. To solve the migration problem, double pigtail stents were deployed within the SEMS and more dedicated FCSEMS with a flare-type, biflanged design (NAGI^®^, Taewoo-Medical, Ilsan, Korea) were developed [29]. Additional lumen-apposing properties were added in these fully covered short metal stents, which creates more apposition forces than just at the flared end [30]. These so-called lumen-apposing metal stents (LAMS) could not only provide a portal for necrotic tissue drainage but could be applied for entero-enteric or entero-biliary anastomosis creation [31]. By the improvement of stent visibility on endoscopic ultrasound (EUS), LAMS insertion could be performed without fluoroscopy [32]. These stents are available in many sizes, ranging from 8 to 20 mm in diameter and 10 to 30 mm in length [33,34]. With the development of an electrocautery-enhanced delivery system, the EUS-guided drainage procedure could be performed in a single step, which eliminates the need for other devices and reduces the procedure time [35,36].

The benefits of LAMS in WON are aiding in the drainage of the debris and easing the endoscopic necrosectomy procedure [5]. There have been many studies directly comparing the efficacy and safety of LAMS and conventional plastic stents (Table 2). Complications after LAMS placement included delayed bleeding and buried LAMS syndrome [37,38]. Data from randomized studies and meta-analyses did not show a significant difference in the overall clinical outcome and adverse events when compared with multiple plastic stents [39,40]. On the contrary, data from multicenter studies showed that the use of LAMS results in higher clinical success after initial drainage and a decreased need for endoscopic necrosectomy [41,42]. Recent data on LAMS as a multigateway approach are promising as it appears to improve the clinical outcome of patients with a large or complex cavity. Due to the high risk of complications in long-term LAMS, the stent should be removed within three weeks of placement if the WON has been resolved [39]. To prevent LAMS occlusion by necrotic debris and distal impaction to the WON cavity, some place another double pigtail stent inside the LAMS, either as primary [38,43] or secondary prophylaxis [44] for LAMS occlusion. In addition, due to the short length, caution should be employed if the distance between the EUS probe and the WON cavity is larger than 1 cm [41].

### 4.2. Endoscopic Necrosectomy

Endoscopic necrosectomy aims to remove the tissue debris and infected material, and open up multiple dead spaces that contain infected material. The procedure could be performed immediately after the initial endoscopic drainage (direct necrosectomy) [54,55] or after a failed clinical response after drainage as a step-up approach [12]. The optimal timing to start endoscopic necrosectomy after the initial procedure ranges from immediately to 48–72 h afterward [39,56]. Generally, endoscopic necrosectomy is recommended only when there is no improvement in clinical response after initial drainage due to a high rate of procedure-related complications [5].

#### 4.2.1. Technical Aspects of Endoscopic Necrosectomy

**Figure 3:**
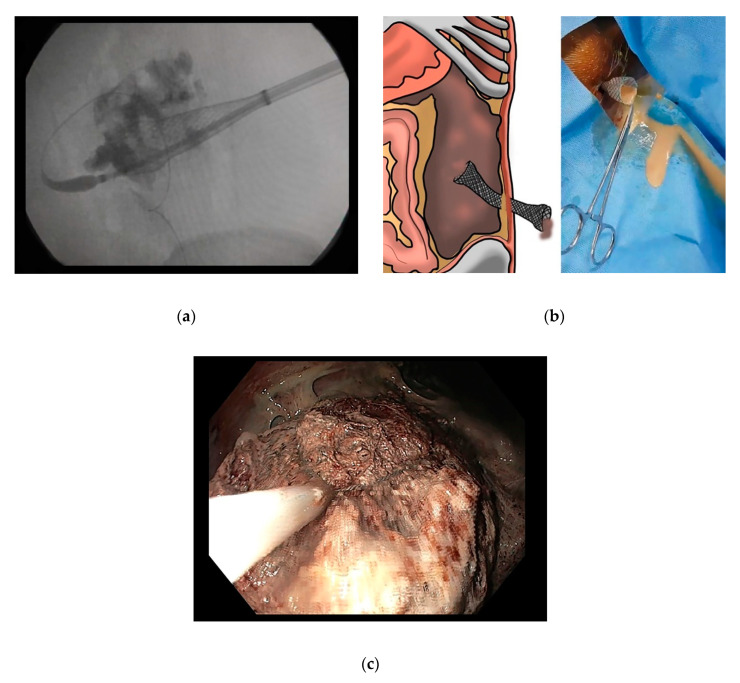
Percutaneous necrosectomy. (**a**,**b**) After percutaneous catheter insertion, a fully covered esophageal stent is placed in the cavity under fluoroscopic guidance. (**c**) Endoscopic necrosectomy can be performed using a small caliber endoscope.

The technique of endoscopic necrosectomy includes mechanical removal and irrigation until pink granulation tissue is seen [57] (Figure 2C,D). The procedure could be performed via the transluminal tract or the percutaneous tract [58]. To aid the necrosectomy, fully covered metallic stents are usually placed after the initial puncture. In the case of transluminal drainage, fully covered esophageal stents or, preferably, lumen-apposing stents are placed [28]; a fully covered esophageal stent can only be used in the transcutaneous approach [59] (Figure 3).

The transluminal procedure is performed by using a flexible gastroscope with a water irrigation system and CO_2_ insufflation, inserted through the fistula tract. Percutaneous necrosectomy can be performed by tract dilation until it is large enough for endoscopic insertion via an overtube or esophageal stent [14]. Tissue debris is mechanically fragmented and removed using a snare, basket, Roth net retriever, tripod/pentapod retriever, or large forceps [25,54,56,57].

#### 4.2.2. Timing of Endoscopic Necrosectomy

In case of early drainage within four weeks after onset, endoscopic debridement can be performed without increasing local complications, regardless of the route of necrosectomy [24]. Interestingly, in comparative studies, perforation after necrosectomy seems to be higher in the late- (>4 weeks) intervention group [24,25]. This indicates that the four-week timing might not be a good general rule of safety for endoscopic procedures and that decisions should be made based on the individual case. However, due to poor encapsulation in the early stage of pancreatitis, endoscopic debridement should be avoided within two weeks of necrosis [5].

The interval between initial stent placement and first necrosectomy is still controversial. Although many endoscopists prefer to delay the first endoscopic necrosectomy until at least a week after the initial stent placement, some prefer to perform direct endoscopic necrosectomy in the first session for early mobilization of the necrotic debris. Concerns over safety and the benefits of early direct endoscopic necrosectomy (DEN) have been reported in a large multicenter study, which showed a decrease in the number of interventions if the endoscopic necrosectomy is performed immediately at the time of LAMS placement [60].

#### 4.2.3. Adjunctive Techniques for Endoscopic Necrosectomy

There are reports of adjunctive techniques that can improve the efficacy of endoscopic necrosectomy. Many studies use a nasocystic tube with irrigation using normal saline [45,47], irrigation during necrosectomy using diluted bacitracin [54], or irrigation with hydrogen peroxide solution [56] and avoidance of acid-suppressing therapy to allow acid digestion of the necrotic debris [5,61]. Despite their widespread use, the benefits of these techniques are not very clear [4]. In cases where initial endoscopic necrosectomy is not effective, additional necrosectomy for the subcavity using the same entry site, so-called “single transluminal gateway transcystic multiple drainages” could be performed [62]. If these methods fail to achieve a clinical response, proceeding to laparoscopic debridement or surgical necrosectomy might be considered [5,63].

**Figure 4:**
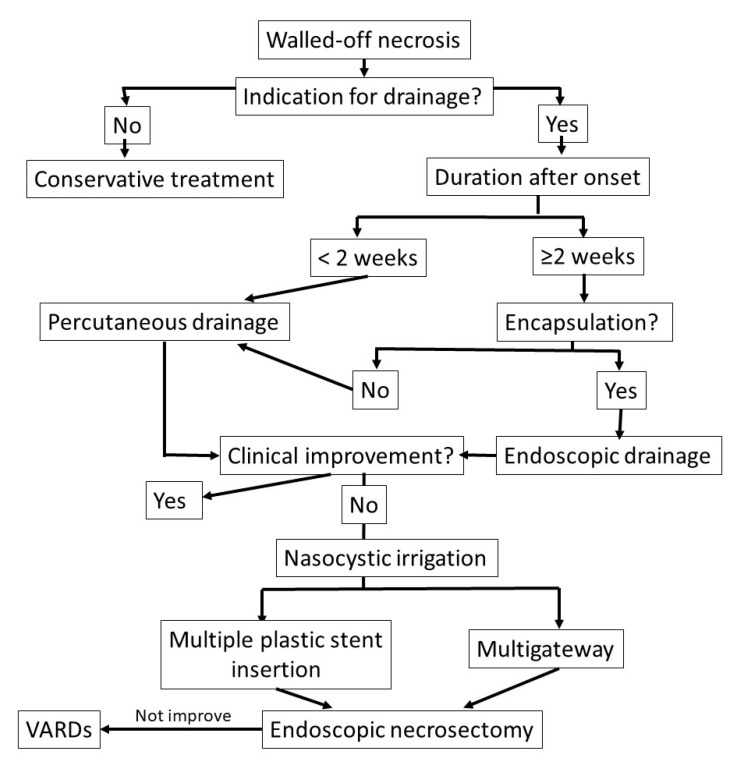
Proposed algorithm for the timely management of walled-off necrosis drainage and necrosectomy.

The proposed algorithm for timely endoscopic drainage and necrosectomy for walled-off necrosis is shown in Figure 4.

## 5. Conclusions

Endoscopic drainage and necrosectomy in walled-off pancreatic necrosis should be performed in a step-up manner. The optimal duration of four weeks was established based on previous studies, but recent studies have pointed to more flexible timing, decided based on individual cases. Early interventions might be performed in the case of walled-off necrosis with the presence of encapsulation, but careful consideration should be given to endoscopic drainage in the very early stage (<2 weeks) since there are limited safety data and encapsulation is not usually present. Several adjunctive methods have been proposed but the benefits are still unclear and the decision should be made on a case-by-case basis.

## Figures and Tables

**Table 1:** Indication for necrosectomy [5].

General Indication for Necrosectomy	Endoscopic Transmural Necrosectomy Preferred	Percutaneous Necrosectomy Preferred
Suspected infection	Centrally located lesion	Paracolic gutter extension
Large amount of necrotic debris	well encapsulation by contrast-enhanced CT	Very early lesion (<2 weeks) or not fully encapsulated
Failed clinical improvement after initial drainage		

**Table 2:** Comparative studies of each type of stents for the treatment of walled-off pancreatic necrosis (WON).

Authors (Year)	Stents	Type of Study	Number of Patients	Outcome	Remarks
Mukai (2015) [45]	DPS versus LAMS (Axios^®^ 15 mm, Nagi^®^ 16 mm, Spaxus^®^ 12 mm)	Retrospective	70	No difference in success but a shorter procedure time with LAMS	Nasocystic irrigation in all cases
Ang (2016) [46]	DPS versus Nagi^®^ 16 mm)	Retrospective	49	DPS associated with higher need for secondary drainage	Both pancreatic pseudocyst and WON included
Bapaye (2017) [47]	DPS versus FCSEMS (Nagi^®^, 16 mm)	Retrospective	133	FCSEMS superior to DPS in terms of clinical success, number of necrosectomies, salvage surgeries, and length of hospital stay	Nasocystic irrigation in all cases
Siddiqui (2017) [48]	DPS versus FCSEMS (10 mm) versus LAMS (Axios^®^ 10,15 mm)	Retrospective	313	FCSEMS and LAMs superior to DPS in efficacy. Fewer procedures are required in LAMS	More acute adverse events in LAMS but fewer stent occlusions or migrations
Abu Dayyeh (2018) [49]	DPS versus FCSEMS (Axios^®^, Nagi^®^, 15, 18, 20 mm)	Retrospective	94	FCSEMS decreases the need for repeated necrosectomy and procedure-related hemorrhage	
Law (2018) [50]	FCSEMS (10 mm) versus LAMS (Axios^®^ 10, 15 mm)	Retrospective	68	Comparable efficacy and safety, but more revisions needed in LAMS	
Lang (2018) [43]	DPS versus LAMS (Axios^®^ 10, 15 mm)	Retrospective	103	Increased complications (bleeding, occlusion) in LAMS	Both pancreatic pseudocyst and WON included
Mohan (2019) [40]	DPS versus LAMS	Meta-analysis	9 studies (737 patients) of LAMS, 7 studies (527 patients) of DPS	Equal clinical outcomes and adverse events in DPS and LAMS	
Bang (2019) [39]	DPS versus LAMS (Axios^®^ 15 mm)	RCT	60	No significant differences in treatment outcome	
Chen (2019) [41]	DPS versus LAMS	Retrospective	189	Higher clinical success, shorter procedure time, lower need for surgery, and lower rate of recurrence in LAMS	
Cho (2019) [51]	DPS versus LAMS (HANARO^®^ 10 mm)	Retrospective	28	No difference in clinical success rate and complications	Pilot study. Included both pseudocyst and WON. New stent with antireflux and antimigration property
Kayal (2020) [42]	DPS versus FCSEMS tubular versus Axios^®^	Historical cohort	58	Higher clinical success in LAMS than FCSEMS and DPS (96.3% vs. 81.8% vs. 77.8%)	Both pancreatic pseudocyst and WON included
Zhu (2020) [52]	DPS versus LAMS (Microtech, 16 mm)	Retrospective	84	Better outcome using LAMS in cases with debris <20%	
Rana (2020) [44]	DPS versus LAMS (Nagi^®^, Plumber^®^, 14, 16 mm)	Retrospective	166	Similar technical success rate, complications, and resolution but shorter time to resolution in LAMS	
Ge (2020) [38]	DPS versus LAMS (Axios^®^ 10, 15 mm)	Retrospective	112	LAMS associated with faster resolution, lower recurrence, and decreased requirement for surgery but higher adverse event rates (bleeding, perforation)	Additional DPS inserted through LAMS
Parsa (2020) [53]	LAMS (Axios^®^) 15 mm versus 20 mm	Retrospective	306	Comparable clinical success and safety but with fewer necrosectomies in larger LAMS	

DPS = double pigtail stent, FCSEMS = fully covered self-expandable tubular stent, LAMS = lumen-apposing metal stent.
